# Preparation and application of methimazole molecularly imprinted polymer based on silver-loaded dendritic fiber-type silica

**DOI:** 10.1039/d1ra00958c

**Published:** 2021-04-12

**Authors:** Chu Hongtao, Chen Jiaqi, Yao Dong, Yu Miao, Lin Qing, Zhang Jingru

**Affiliations:** College of Chemistry and Chemical Engineering, Qiqihaer University Qiqihaer 161006 China lange1979@163.com

## Abstract

Dendritic fiber-type silica (KCC-1) has attracted the attention of researchers because of its unique three-dimensional radial structure and high specific surface area. Its highly modified surface allows it to be used in catalysis, adsorption, biomedicine, and other fields. Nano-precious metals (NPs) have several excellent chemical properties, but their stability limits their applications. Dendritic fibrous silica (Ag NPs/KCC-1) loaded with silver nanoparticles was prepared *via* the microemulsion method using Ag NPs/KCC-1 as the carrier, methimazole as the template molecule, and a surface imprinting method to prepare sulfhydryl imidazole molecularly imprinted polymer. By characterizing the polymer, it is determined that the polymer has a regular morphology and large specific surface area. The obtained experimental results show that the polymer has a high adsorption capacity (10.35 mg g^−1^) and good selectivity. It is used as a solid-phase extraction filler and, when combined with high-performance liquid chromatography, to detect methimazole in chicken tissue. The recovery rate reaches 87.5–94.4%.

## Abstract 2

Dendritic fiber-type silica (KCC-1) has attracted the attention of researchers because of its unique three-dimensional radial structure and high specific surface area.

## Graphical abstract

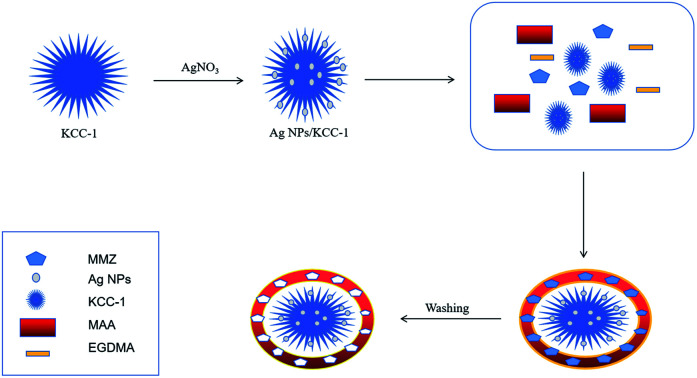

## Introduction

Methimazole is a clinical anti-thyroid drug that enhances protein metabolism and increases lean meat rate. Thus, it is added to animal feed by illegal traders, which results in excessive content in animal food.^1,2^ After humans consume such animal foods, it causes the continuous accumulation of methimazole in the body and results in disease and even death.^3,4^ With an increase in people's awareness of food safety, the detection of methimazole residues has attracted increased attention. Hence, developing an efficient and simple method for detecting methimazole is practically significant.

Molecular imprinting technology is an essential method for developing highly selective enrichment materials. The most notable feature of molecular imprinting technology is its specific recognition, which can identify and enrich target molecules from complex matrices; this approach is often used to selectively enrich materials that are used as samples and for the preparation of molecularly imprinted polymers.^5–7^ However, traditional molecularly imprinted polymers have a large internal core mass transfer resistance, and the active site is embedded too deeply, which makes it difficult to elute the template molecules and results in poor reusability. To solve this problem, the surface imprinting method initiates the polymerization reaction on the surface of the carrier so that the binding site is located on the surface. Thus, the imprinted polymer prepared *via* the surface polymerization method has a fast rate of elution and adsorption of template molecules and required a short period to reach the adsorption equilibrium. The specific recognition ability is strong, and with the carrier as the support, the shape of the imprinted polymer is more regular, the mechanical stability is improved, and the service life is longer.^8,9^ Huang *et al.*^10^ have used nicotine as a template molecule to prepare an imprinted polymer on the surface of silica and used it as a gas chromatography stationary phase to test its retention capacity and to remove nicotine from environmental smoke. Feng *et al.*^11^ have used molecular imprinting solid phase extraction high-performance liquid chromatography (HPLC) to detect the residues of tetracycline drugs in food. The prepared molecular-imprinted solid-phase extraction column can capture four tetracycline drugs at the same time and has a high adsorption capacity and recovery rate and can be reused at least 10 times.

Dendritic fiber-type silica (KCC-1) is a new type of nanoporous silica with a high specific surface area and excellent physical and chemical properties.^12–14^ KCC-1 has shown its broad prospects in energy,^15,16^ environmental governance,^17,18^ catalysis,^19,20^ and sensors.^21,22^ Because of the abundant silanol groups on the surface of KCC-1 and highly porous surface, it is a good carrier for surface molecularly imprinted polymers. There are no reports on the use of KCC-1 as a surface imprinting body.

Nanosilver is widely used in the fields of catalysts and antistatic and medical antibacterial materials because of its several surface active points, high electrical conductivity, and antibacterial properties. However, the gathering of nanosilver in the application process affects its application effect and performance.^23^ KCC-1 can be used as a carrier template for loading nanosilver because of its special pore structure. The pore structure improves nanosilver stability.^24^ At the same time, the special force formed by silver and sulfhydryl compounds increases resistance to sulfhydryl compounds, adsorption capacity, and adsorption rate.

This study uses methimazole as the template molecule; KCC-1 is used for the first time as the carrier of molecular imprinting technology, and nanosilver particles are loaded in it to enhance its antibacterial properties and adsorption rate and to achieve nanosilver-supported methimazole molecularly imprinted polymerization. It is utilized as a solid-phase extraction filler and, combined with HPLC, to enrich and detect methimazole in chicken tissue.

## Experiment

### Materials

Cetyl pyridinium bromide, urea, 3-aminopropylethoxysilane (APTES), ethylene glycol dimethacrylate (EGDMA) (Aladdin Reagent Co., Ltd.), *N*-pentanol, cyclohexane, TEOS (Tianjin Kermel Chemical Reagent Co., Ltd.), 2-mercaptoimidazole (MYZ), *N*-methylthiourea (Metu), methimazole (MMZ) (Shanghai McLean Biochemical Co., Ltd.), silver nitrate, sodium hydrogen borate, azobisisobutyronitrile (AIBN) (Sinopharm Chemical Reagent Co., Ltd.), beef extract, bacterial peptone, and technical agar powder (Guangdong Huankai Microbial Technology Co., Ltd.) were used. The fresh chicken samples (chicken liver and chicken) were obtained from the poultry slaughtered the previous day from a commercial establishment in Qiqihar, Heilongjiang. All aqueous solutions used for polymerization, swelling, and adsorption studies were prepared with deionized water.

### Preparation of dendritic silica KCC-1

Measure 3.0 mL of *n*-pentanol and 60 mL of cyclohexane in the flask and add 5.4 mL of ethyl orthosilicate under stirring. Weigh 2 g of cetylpyridinium bromide and 1.2 g of urea in 60 mL of deionized water, introduce the solution into the flask, stir at 35 °C for 30 min, transfer to the reactor, heat at 140 °C for 6 h, and cool to room temperature. After centrifugal separation at 5000 rpm and washing with ethyl acetate and ethanol three times sequentially, the obtained solid was dried at 60 °C for 12 h, and the final product was calcined in a muffle furnace at 550 °C for 6 h to obtain KCC-1.

### Preparation of Ag NPs/KCC-1

A total of 1.0 g of KCC-1 and 0.3 g of 3-APTES were mixed in 100 mL of toluene, stirred, and refluxed under N_2_ atmosphere at 80 °C for 12 h. The obtained amino-functionalized KCC-1 (H_2_N-KCC-1) was washed sequentially with chloroform, dichloromethane, and ethanol, and dried in a vacuum. A total of 0.5 g of H_2_N-KCC-1 was ultrasonically dispersed in 50 mL of H_2_O. Then, 0.1 g of AgNO_3_ was added and stirred for 30 min; NaBH_4_ solution was added dropwise, and the obtained product was vacuum dried to obtain Ag NPs/KCC-1.

### Preparation of methimazole molecularly imprinted polymer

Add MMZ (8.4 mg), MAA (51.12 μL), EGDMA (282.9 μL), AIBN (13 mg), and 0.5 g of Ag NPs/KCC-1 to 20 mL of acetonitrile, stir for 10 min, and purge with N_2_ for 15 min at 60 °C to initiate polymerization; methanol–acetic acid (9 : 1, v/v) as the eluent after centrifugation and elute the polymer by Soxhlet extraction for 48 h until the template molecule is completely removed; the obtained solid is washed with water to neutrality.

As a control, without adding MMZ, the preparation process was the same as above, and the non-imprinted polymer Ag NPs/KCC-1/NIP was prepared. To investigate the advantages of Ag NPs, the imprinted polymer KCC-1/MIP with KCC-1 as the carrier was also made. To investigate the advantages of the KCC-1 carrier, the imprinted polymer *N*-MIP without KCC-1 was also prepared.

### Sample characterization

The samples were characterized by field emission scanning electron microscopy (JSM-7800F, Jeol Ltd, Japan), transmission electron microscopy, X-ray diffractometry (D8, BRUKER-AXS Ltd., Germany), and X-ray photoelectron spectroscopy (Thermo, USA).

### Adsorption experiment

A total of 10.0 mg of Ag NPs/KCC-1/MIP, Ag NPs/KCC-1/NIP, KCC-1/MIP, and *N*-MIP was added to 10 mL of MMZ methanol solutions of different concentrations (10–150 mg L^−1^). The mixture was allowed to stand at room temperature for 12 h; the supernatant was filtered, and the concentration of MMZ was measured at 254 nm using ultraviolet-visible spectrophotometry. The adsorption capacity is calculated based on the formula:
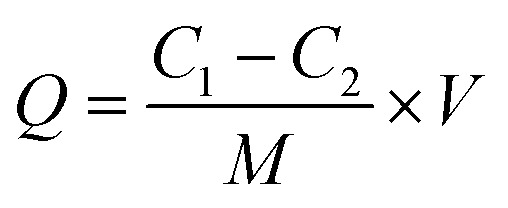
where *Q* (mg g^−1^) is the adsorption capacity; *C*_1_ and *C*_2_ (mg L^−1^) are the initial concentration and final concentration, respectively; *V* (mL) is the volume of the solution; *M* (mg) is the mass of added adsorbent.

The adsorption kinetic data were tested as follows. A total of 10.0 mg of Ag NPs/KCC-1/MIP, KCC-1/MIP, and *N*-MIP was dispersed in 10 mL of the MMZ methanol solution (100 mg L^−1^). The obtained solution is allowed to stand at room temperature for different periods (*i.e.*, 5, 10, 20, 30, 40, 60, 100, and 120 min). Then, the supernatant is filtered. Spectrophotometry (254 nm detection wavelength) is used to measure the adsorption amount of the adsorbent.

The specific adsorption performance of Ag NPs/KCC-1/MIP was investigated. A total of 10.0 mg of Ag NPs/KCC-1/MIP and Ag NPs/KCC-1/NIP was dispersed in methimazole, *N*-methylthiourea, 2-mercaptoimidazole (the concentration of each is 100 mg L^−1^), and methanol solution (10 mL). The mixture was allowed to stand at room temperature for 2 h; the supernatant was filtered, and the adsorption capacity in different solutions was calculated.

The reproducibility of the adsorption capacity of Ag NPs/KCC-1/MIP was investigated. A total of 10.0 mg of Ag NPs/KCC-1/MIP was dispersed in 10 mL of the MMZ methanol solution with a concentration of 100 mg L^−1^. The mixture was allowed to stand at room temperature for 2 h, and the supernatant was filtered; then, the adsorption capacity was calculated. After centrifugation, methanol : acetic acid (9 : 1) was used as the eluent, and the polymer was eluted by Soxhlet extraction for 48 h to completely remove the template molecule; the obtained solid was washed with water to neutrality. This step is repeated six times to calculate the adsorption capacity.

### Antibacterial experiment

For the minimum inhibitory concentration (MIC) test, fresh *E. coli* suspension is diluted to the concentration of 1.5 × 10^8^ CFU mL^−1^ [based on prior experience, the bacterial suspension absorbs at the wavelength of 600 nm (OD_600_)]. When the UV absorbance value is 0.5, the concentration of bacterial suspension is 1.5 × 10^8^ CFU mL^−1^. A certain amount of sample is used to prepare a series of concentration sample solutions and a set of blank solutions. A total of 1 mL of the diluted bacterial suspension, 1 mL of a certain concentration of sample solution, and 98 mL of liquid medium NB are added to a conical flask. The final concentration of the bacterial suspension was 1.5 × 10^6^ CFU mL^−1^; the final concentration of the sample solution was 10, 20, 30, 40, 50, 60, and 70 mg L^−1^, and a set of blank control samples was used. The abovementioned mixture was incubated at 37 °C on a shaker for 24 h. Its OD_600_ value is tested at regular intervals. The minimum concentration at which the OD_600_ of the mixed solution does not change after 24 h is the MIC.

For the minimum bactericidal concentration (MBC) test, the abovementioned groups of mixed solutions whose OD_600_ does not change after 24 h are applied to the solid medium and placed in a 37 °C incubator for 24 h to observe the growth of colonies. The minimum concentration without colony growth is the MBC.

### Analysis of real samples

For sample pretreatment, fresh chicken samples (chicken liver and chicken) are used as biological samples for matrix addition and recovery experiments. A total of 5 g of test samples are weighed, crushed, and placed in a 50 mL polypropylene centrifuge tube. The spiked concentrations are 0.01, 0.02, and 0.05 μg g^−1^ MMZ, respectively. After standing in the dark for 10 min, 10 mL of acetonitrile was added to extract the analyte and precipitate the protein. The mixture is centrifuged at 3500 rpm at 4 °C for 5 min, and the extraction of residue is repeated using the same procedure. The two supernatants were combined and mixed with 30 mL of acetonitrile saturated *n*-hexane to remove fat in the sample. After shaking for 3 min, the acetonitrile phase was separated, dried over anhydrous sodium sulfate, and the residue was washed with 2 mL of acetonitrile. The product was rotary-evaporated at 40 °C; the dried acetonitrile extract was dissolved in ethanol and transferred to a 100 mL volumetric flask; the volume was constant; then, the product was analyzed by the developed molecularly imprinted solid-phase extraction (MISPE) and HPLC methods.

For the solid phase extraction, 100 mg of Ag NPs/KCC-1/MIP is added into a 3 mL SPE empty column tube. Sieve plates are placed on both sides to fix it, and a MISPE small column is prepared using 5 mL of water and activated using 10 mL of methanol. A total of 3 mL of the test solution is used, and the sample is loaded at the flow rate of 0.4 mL min^−1^. The sample is rinsed with 5 mL of water, and the adsorbed solution is eluted with 4 mL of a glacial acetic acid–methanol solution (1 : 9, v/v). Methimazole is added to the column; the eluent is collected and blow-dried with nitrogen. The obtained product is dissolved in the mobile phase, filtered with the membrane, and the HPLC measurement is performed.

HPLC parameters: chromatographic column: (4.6 mm × 200 mm, Hypersil ODS2 5 μm, DIONEX); mobile phase: methanol–water (9 : 1, v/v). Flow rate: 1.0 mL min^−1^; detection wavelength: 254 nm; injection volume: 20 μL.

## Results and discussion

### Sample characterization

#### Ag NPs/KCC-1 XRD analysis

**Fig. 1:**
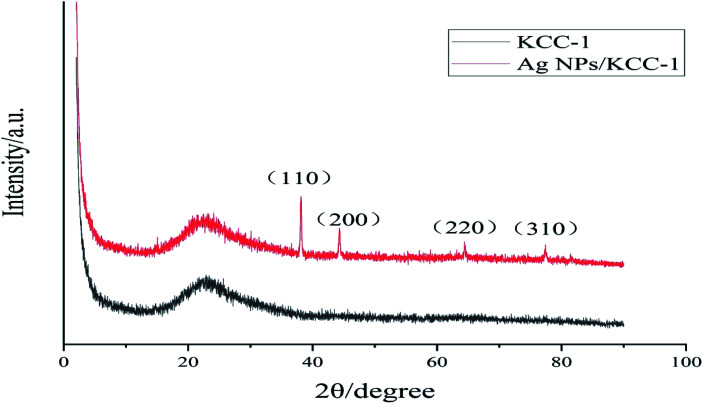
XRD spectra of KCC-1 and Ag NPs/KCC-1.

Fig. 1 shows the XRD spectra of KCC-1 and Ag NPs/KCC-1. Both compounds show a strong and wide diffraction peak at 2*θ* = 23.06°, which is the characteristic peak of silica. This result indicates that KCC-1 is an amorphous silica compound. Compared with the spectrum of KCC-1, the spectrum of Ag NPs/KCC-1 exhibits new peaks at 38.13°, 46.16°, 64.40°, and 77.39°, which correspond to the (111), (200), (220), and (311) crystal planes of cubic Ag NPs. No other peaks are found in the two curves. Therefore, the surface of samples has high purity, and Ag exists in a simple form. These results confirm the successful loading of Ag NPs onto KCC-1.

#### Ag NPs/KCC-1 XPS analysis

**Fig. 2:**
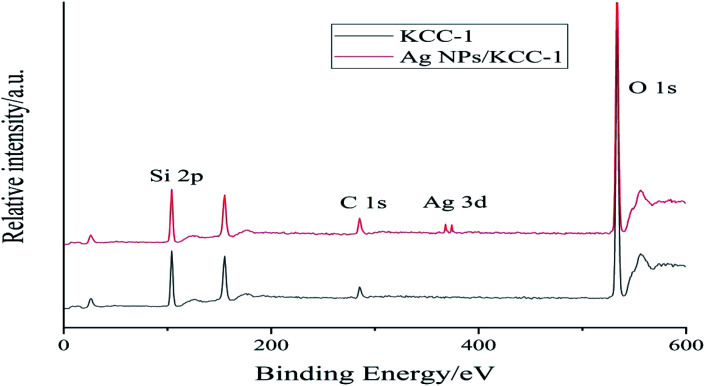
XPS spectra of KCC-1 and Ag NPs/KCC-1.

Fig. 2 shows the XPS spectra of KCC-1 and Ag NPs/KCC-1. The binding energies of Si 2p and O 1s of KCC-1 appear at 104.30 and 537.23 eV, respectively. The XPS spectrum of Ag NPs/KCC-1 clearly shows the binding energies of Ag 3d_5/2_ and Ag 3d_3/2_ at 367.60 and 373.65 eV, respectively, and the absence of changes in the original KCC-1 peaks. Ag on the KCC-1 surface exists in the form of elemental Ag; TEM is used to confirm the successful loading of Ag NPs and the successful preparation of composite materials.

#### FTIR analysis

**Fig. 3:**
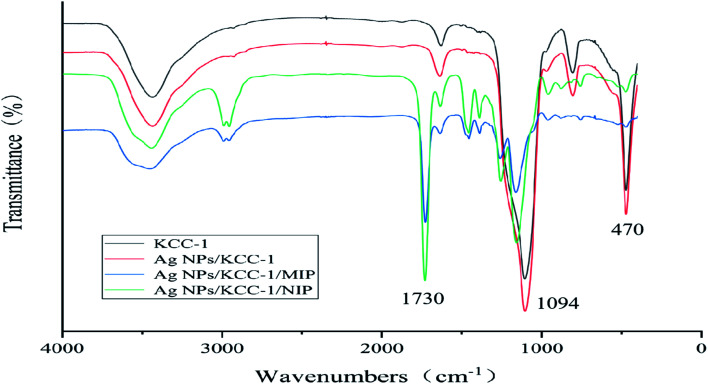
FT-IR spectra of KCC-1, Ag NPs/KCC-1, Ag NPs/KCC-1/MIP, and Ag NPs/KCC-1/NIP.

The surface groups of KCC-1, Ag NPs/KCC-1, Ag NPs/KCC-1/MIP, and Ag NPs/KCC-1/NIP were characterized *via* Fourier-transform infrared spectroscopy (FTIR) (Fig. 3). Significant bands at 470 cm^−1^ (Si–O–Si stretching vibration) and 1094 cm^−1^ (Si–OH stretching vibration) were observed in the spectra of the four samples. Both for KCC-1 and Ag NPs/KCC-1, there was no significant change in the band, which indicated that silver existed in elemental form. Additionally, in the spectra of Ag NPs/KCC-1/MIP and Ag NPs/KCC-1/NIP, 1730 cm^−1^ (C=O MAA stretching vibration) and 1157 cm^−1^ (C–O EGDMA stretching vibration) bands can be observed. The infrared characteristic peaks directly confirm that the functional monomer MAA and cross-linking agent EGDMA are involved in the polymerization, and the molecularly imprinted polymer is successfully synthesized. The infrared spectra of the two are not significantly different, which indicates that the template molecule eluted completely from Ag NPs/KCC-1/MIP.

#### Scanning electron microscopy and EDS characterization

**Fig. 4:**
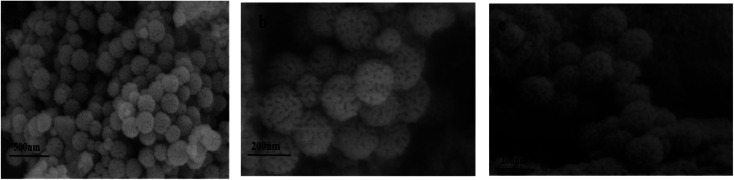
SEM images of the samples (a) KCC-1, (b) Ag NPs/KCC-1, and (c) Ag NPs/KCC-1/MIP.

Fig. 4 shows the SEM images of KCC-1, Ag NPs/KCC-1, and Ag NPs/KCC-1/MIP. KCC-1 is radial silica, and the fibrous pore structure on its surface can be observed. In the SEM image of Ag NPs/KCC-1, silver nanoparticles are not observed on the surface, which does not significantly differ from KCC-1 because Ag NPs in the pore structure cannot be observed. Then, TEM measurements are performed. Compared with KCC-1 and Ag NPs/KCC-1, the uneven molecularly imprinted layer on the surface of Ag NPs/KCC-1/MIP can be observed (Table 1).

**Table 1:** EDS spectrum of Ag NPs/KCC-1

Element	Conc%	Atomic%
O	43.782	52.889
Si	45.564	42.452
Ag	10.654	4.659
Total	100%	100%

**Fig. 5:**
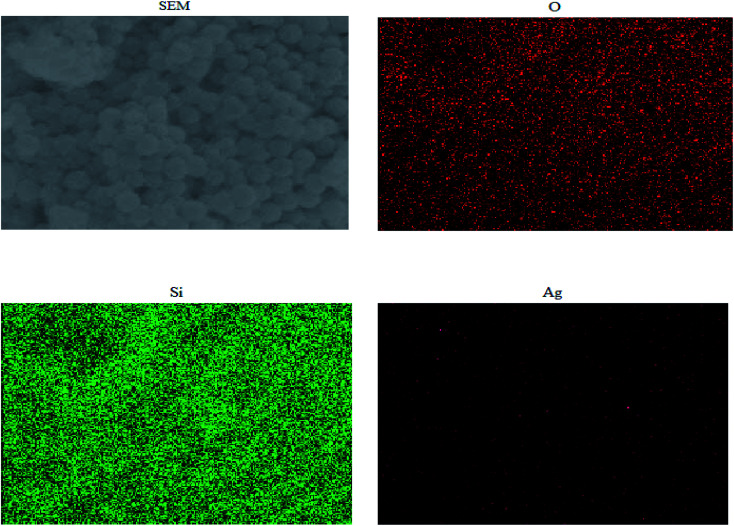
Mapping analysis of Ag NPs/KCC-1.

**Fig. 6:**
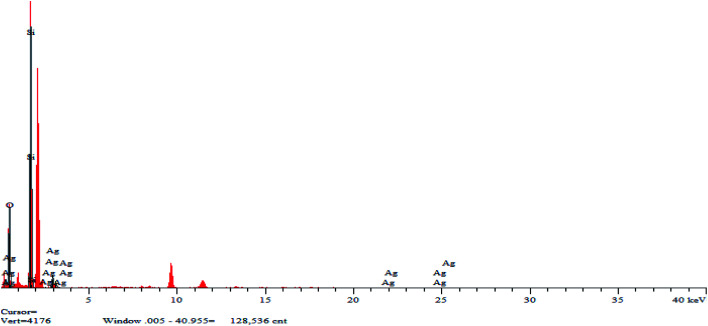
EDS spectrum of Ag NPs/KCC-1.

Fig. 5 shows the mapping analysis image of Ag NPs/KCC-1. The surface of Ag NPs/KCC-1 was selected for testing. Different elements are indicated by different colors; specifically, oxygen is red, silicon is green, and silver is pink. Fig. 6 shows the EDX analysis of Ag NPs/KCC-1. The obtained results show that a large amount of silicon and oxygen is distributed on the surface of Ag NPs/KCC-1, the mass fractions of which are 43.782% and 45.564%, respectively. The mass fraction of silver is 10.654%.

#### TEM characterization

**Fig. 7:**
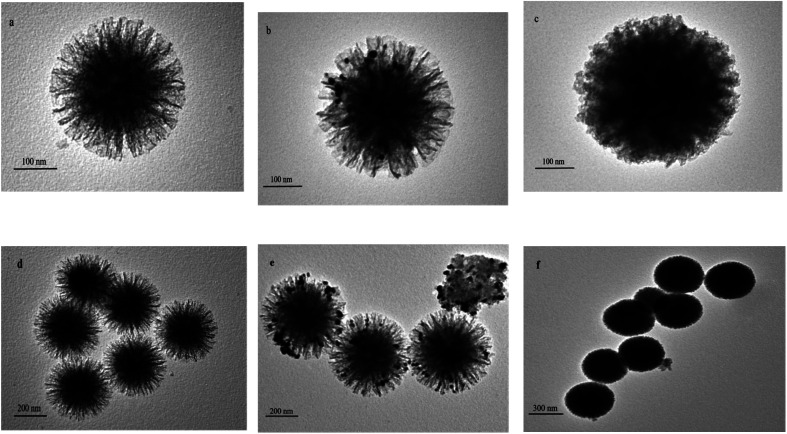
TEM images of the samples (a and d) KCC-1, (b and e) Ag NPs/KCC-1, and (c and f) Ag NPs/KCC-1/MIP.

Fig. 7 shows the TEM images of KCC-1, Ag NPs/KCC-1, and Ag NPs/KCC-1/MIP. Fig. 7(a) and (d) shows that the particle size of KCC-1 is approximately 300 nm, and its uniform fibrous pore structure can be observed. In the TEM image of Ag NPs/KCC-1 in Fig. 7(b and e), it can be observed that the fibrous pore structure contains silver nanoparticles, which indicates the successful loading of Ag NPs. On the basis of Ag NPs/KCC-1, Ag NPs/KCC-1/MIP can observe a clear circle of molecularly imprinted layer, which has a larger particle size than that of KCC-1, and the particle size is approximately 400 nm.

#### BET characterization

**Fig. 8:**
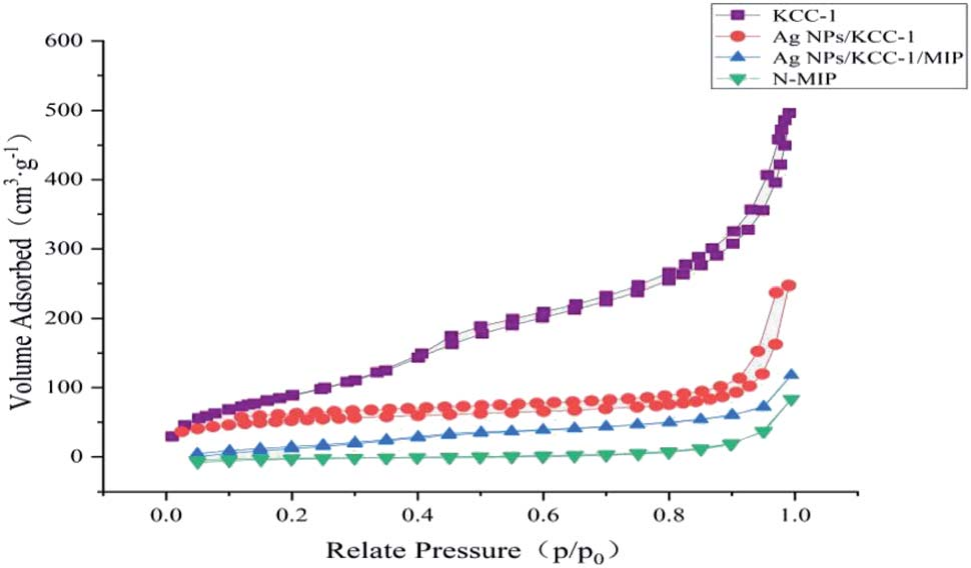
Nitrogen adsorption–desorption isotherms of KCC-1, *N*-MIP, Ag NPs/KCC-1, and Ag NPs/KCC-1/MIP.

The specific surface area of KCC-1, *N*-MIP, Ag NPs/KCC-1, and Ag NPs/KCC-1/MIP was tested using the nitrogen adsorption–desorption isotherm method. Fig. 8 shows that the specific surface area of dendritic fibrous silica KCC-1 is as high as 490.82 m^2^ g^−1^, which decreases to 275.65 m^2^ g^−1^ after loading Ag NPs, and decreases to 151.32 m^2^ g^−1^ after loading the imprinting layer; however, it remains considerably higher than the value of 46.82 m^2^ g^−1^ for *N*-MIP.

#### Thermogravimetric analysis (TGA)

**Fig. 9:**
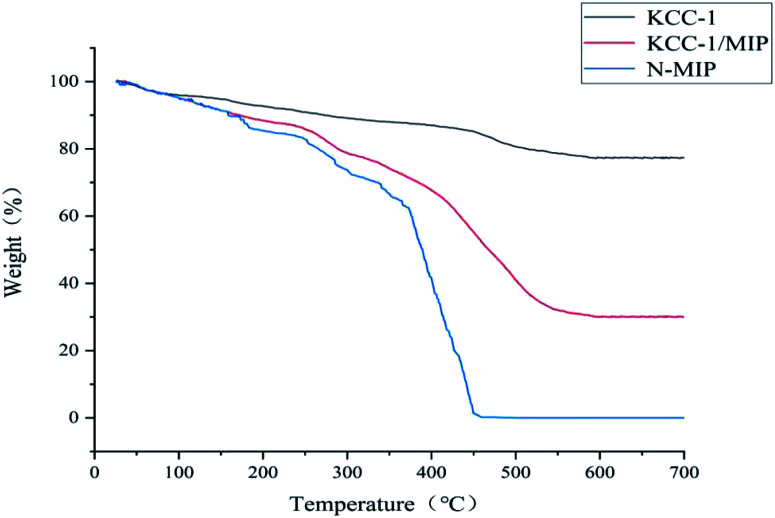
TGA curves of KCC-1, KCC-1/MIP, and *N*-MIP.

Fig. 9 shows the TGA of KCC-1, KCC-1/MIP, and *N*-MIP. During the temperature increase from 100 °C to 300 °C, all three samples experienced a slight weight loss because of the evaporation of residual moisture. The weight loss from 300 °C to 450 °C is due to the decomposition of the molecularly imprinted polymer matrix. *N*-MIP completely decomposes at 450 °C, and its mass loss is attributed to the internal matrix KCC-1, which does not have high thermal stability, which causes *N*-MIP to decompose more easily than KCC-1/MIP at high temperatures. At the same time, the residual amount of KCC-1/MIP is higher, and the decomposition temperature is much higher than that of *N*-MIP, which indicates that KCC-1/MIP with internal matrix KCC-1 has higher thermal stability.

### Adsorption test

#### Thermodynamic adsorption test

**Fig. 10:**
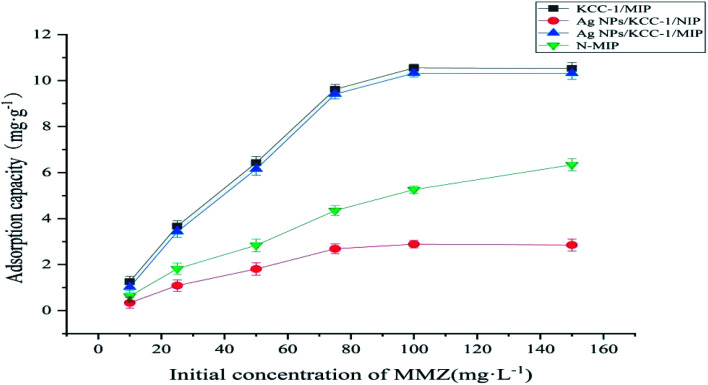
Thermodynamic adsorption curves of KCC-1/MIP, Ag NPs/KCC-1/MIP, Ag NPs/KCC-1/NIP, and *N*-MIP.

The static adsorption of KCC-1/MIP, Ag NPs/KCC-1/MIP, Ag NPs/KCC-1/NIP, and *N*-MIP to MMZ was tested under different MMZ concentrations (10–150 mg L^−1^). Fig. 10 shows the result. The abovementioned figure shows that the adsorption capacity of all tested polymers rapidly increases with an increase in the MMZ concentration. Under the same concentration, the adsorption capacity of MMZ on Ag NPs/KCC-1/MIP and KCC-1/MIP is considerably higher than that of *N*-MIP. Generally, the absorption capacity of surface-imprinted polymer is lower than that of bulk polymerized imprinting material because imprinting holes only exist on the surface of the matrix material, and their total amount is small. Contrary to what has been reported, the special fibrous structure of the carrier material KCC-1 has a large surface area; thus, there are considerably more imprinted holes on Ag NPs/KCC-1/MIP and KCC-1/MIP. Compared with *N*-MIP, it has a higher bonding performance. The adsorption capacity of Ag NPs/KCC-1/MIP (10.35 mg g^−1^) is slightly lower than that of KCC-1/MIP (10.55 mg g^−1^) because Ag NPs occupies part of the pore structure and reduces the adsorption sites. Additionally, the binding capacity of MMZ on Ag NPs/KCC-1/MIP is 3.6 times that of Ag NPs/KCC-1/NIP (2.85 mg g^−1^), which is attributed to the specific recognition site of MMZ formed during the imprinting process. Ag NPs/KCC-1/NIP does not have such a site.

#### Kinetic adsorption test

**Fig. 11:**
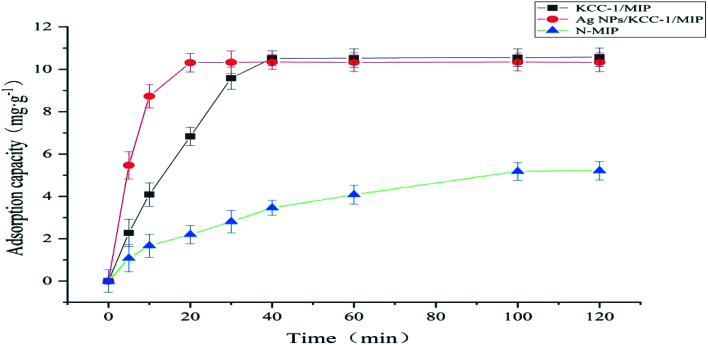
Kinetic adsorption curves of KCC-1/MIP, Ag NPs/KCC-1/MIP, and *N*-MIP.

To explore the adsorption rate, the kinetic adsorption curves of KCC-1/MIP, Ag NPs/KCC-1/MIP, and *N*-MIP on 100 mg L^−1^ MMZ were drawn. Compared with *N*-MIP with an equilibrium time of 100 min, KCC-1/MIP and Ag NPs/KCC-1/MIP showed faster adsorption rate and equilibrium time. This can be summarized as follows. Because of the special pore structure and large specific surface area of KCC-1, more MMZ recognition sites are located on the surface of the fibrous silica sphere and pore structure; hence, the target analyte can easily diffuse to the recognition site, which reduces the mass transfer resistance. At the same time, because of the special chemical force between Ag NPs and sulfhydryl compounds in KCC-1 pores, Ag NPs/KCC-1/MIP (20 min) reaches adsorption equilibrium faster than KCC-1/MIP (40 min). By contrast, *N*-MIP is prepared *via* bulk polymerization, in which a large number of imprinting sites are located in the inner region, which results in difficult adsorption and slow mass transfer (Fig. 11).

#### Selective adsorption experiment

**Fig. 12:**
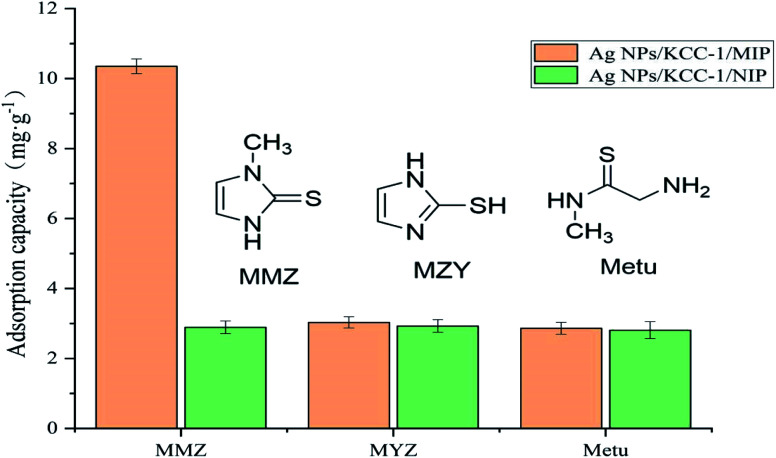
Selective adsorption of Ag NPs/KCC-1/MIP to MMZ, MYZ, and Metu.

To evaluate the selective adsorption performance of the prepared polymer to MMZ, a comparative adsorption experiment of MZY and Metu was performed. As shown in the Fig. 12, MZY and Metu have similar structures to MMZ. The adsorption capacity of Ag NPs/KCC-1/MIP for the target component MMZ is significantly higher than for other structural analogs, and it is also greater than that of Ag NPs/KCC-1/NIP. Conversely, the adsorption capacity of Ag NPs/KCC-1/NIP to the three structural analogs is low, and there is no significant difference. The partition coefficients of Ag NPs/KCC-1/MIP to MMZ, MZY, and Metu are 11.54, 3.12, 2.94, respectively, and the imprinting factors are 3.58, 1.03, and 1.02, respectively, which indicates that Ag NPs/KCC-1/MIP has the most selective adsorption to MMZ. The adsorption capacity of Ag NPs/KCC-1/MIP to MMZ is much greater than that of MZY and Metu, mainly because there are imprinting holes in Ag NPs/KCC-1/MIP that only match the structure of MMZ. For this type of imprinting, the pores do not match the structure of the other two substances. Ag NPs/KCC-1/NIP does not have the imprinted pores of the abovementioned three substances. The adsorption process of the substrate is mainly physical adsorption; thus, there is no clear difference in the adsorption capacity.

#### Repeatability experiment

**Fig. 13:**
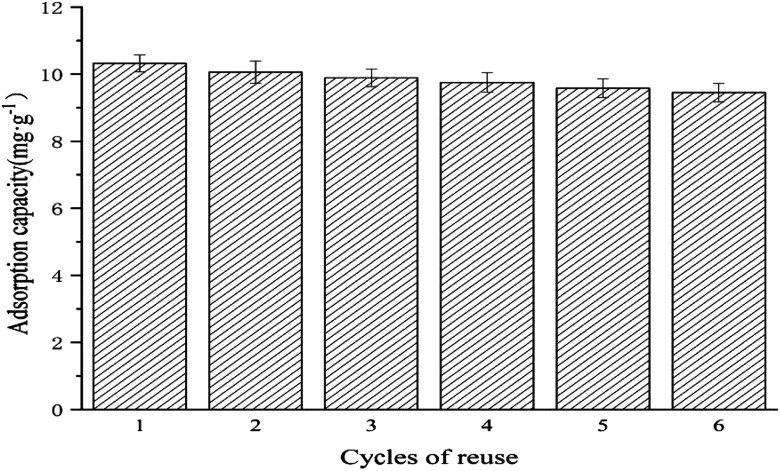
Repeatability experiment for Ag NPs/KCC-1/MIP toward MMZ.

After the prepared molecularly imprinted polymer is applied to several cycles of adsorption and desorption, the polymer still has a specific adsorption capacity for the target molecule. Fig. 13 shows the results. To evaluate this, the adsorption of the methanol solution of MMZ (100 mg L^−1^) by Ag NPs/KCC-1/MIP is performed, and elution with methanol : acetic acid (volume ratio 9 : 1) as the eluent is performed until the eluent can no longer be detected by the template molecule. The obtained results showed that the adsorption amount of the molecularly imprinted polymer to the template molecule MMZ insignificantly changed, and the adsorption amount after six cycles was still 91.57% of the original value, which indicated that the molecularly imprinted polymer prepared by this method had a relatively stable rigid structure. During repeated adsorption and elution, the loss of adsorption sites is less, and Ag NPs/KCC-1/MIP can be repeatedly used and has good reproducibility for the adsorption of MMZ.

### Antibacterial test

**Fig. 14:**
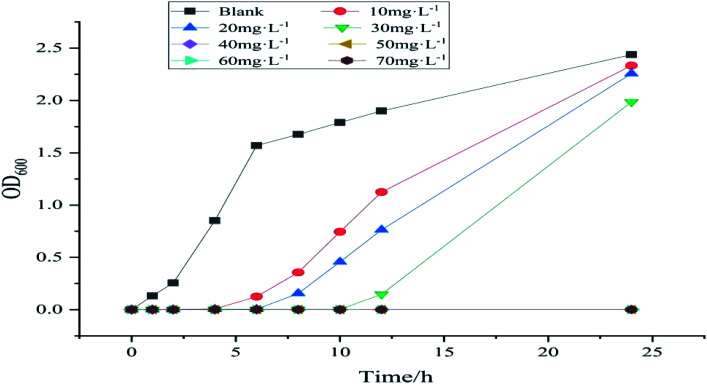
MIC curves of different concentrations of Ag NPs/KCC-1/MIP.

Given that MMZ may exist in several biological samples, to meet the reusability in various environments, its antibacterial properties are investigated. Fig. 14 shows the MIC curves of samples with different concentrations. In this figure, the concentration of bacterial suspension of the blank group of samples increased over time, whereas the OD_600_ of the bacterial suspension of the other group of samples at 2–6 h was almost 0, and the concentration hardly increased. This result shows that Ag NPs/KCC-1/MIP has an antibacterial effect. With an increase in time, the bacterial concentration of samples below 40 mg L^−1^ increases, and the bacterial concentration increases to the same level as that of the blank group after 24 h. The bacterial concentration of samples greater than or equal to 50 mg L^−1^ within 24 h remains almost unchanged. Thus, it can be concluded that the MIC of the sample is 40–50 mg L^−1^. Because the silver content of the sample is 10.65%, the MIC of the silver in the sample is 4.26–5.32 mg L^−1^.

**Fig. 15:**
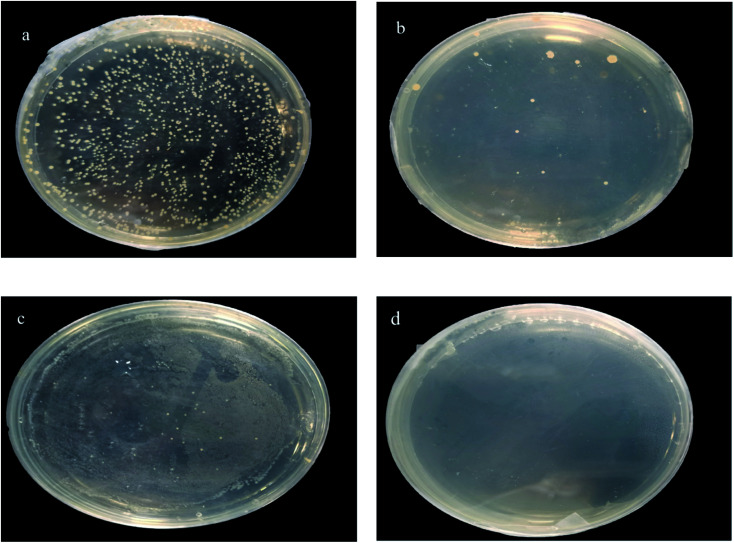
MBC pictures of samples with different concentrations (a) 40 mg L^−1^, (b) 50 mg L^−1^, (c) 60 mg L^−1^, and (d) 70 mg L^−1^.

Fig. 15 shows the MBC experiment for samples with different concentrations. After 24 h of reproduction, samples with 40, 50, and 60 mg L^−1^ had bacterial colonies; only the 70 mg L^−1^ samples did not have bacterial colonies. Therefore, it is determined that MBC is 70 mg L^−1^. The MBC of silver in this sample is 7.45 mg L^−1^. It is reported in the literature that MIC and MBC of pure silver with a diameter of 7 nm are 6.5 and 12.5 mg L^−1^.^25^ MBC is 7.45 mg L^−1^, and the antibacterial effect is higher than that reported in the literature.

### Method establishment

Combine MISPE with the HPLC method to establish a fast and analytical method for the determination of MMZ. Prepare a 5–50 μg L^−1^ MMZ standard solution, perform chromatographic analysis, and inject the solution five times in parallel. Draw a standard curve with the concentration of the MMZ standard solution as the abscissa and the chromatographic peak area as the ordinate. The obtained results show that within the investigated range, the MMZ concentration and the chromatographic peak area (*A*) have a good linear relationship; the linear regression equation is *A* = 4576.3*c* + 36.07, and the linear correlation coefficient is *R*^2^ = 0.9997. The detection limit is 0.52 μg L^−1^ (S/N = 3), and the quantification limit is 1.62 μg L^−1^ (S/N = 10).

### Analysis of actual samples

The prepared imprinted polymer was used as the solid-phase extraction filler to prepare MISPE cartridges. The chicken breast and chicken liver samples were analyzed by HPLC. No MMZ was detected in the two samples. The samples were spiked and analyzed. MISPE cartridges were used to perform solid-phase extraction on the chicken breast and chicken liver samples. Table 2 shows the results. At the addition levels of 0.01, 0.02, and 0.03 μg g^−1^, the recovery rates of MMZ in different sample matrices were measured to be in the range of 87.5–94.4%, and the relative standard deviation was less than 4.6% (Table 3).

**Table 2:** Distribution coefficients and imprinting factors of Ag NPs/KCC-1/MIP and Ag NPs/KCC-1/NIP

Compound	Adsorption capacity *Q* (mg g^−1^)		Distribution coefficient *K*_d_ (mL g^−1^)		Imprinting factors *α*
	Ag NPs/KCC-1/MIP	Ag NPs/KCC-1/NIP	Ag NPs/KCC-1/MIP	Ag NPs/KCC-1/NIP	
MMZ	10.35	2.89	11.54	2.97	3.58
MYZ	3.03	2.93	3.12	3.01	1.03
Metu	2.86	2.81	2.94	2.89	1.02

**Table 3:** Results of the standard addition recovery experiment (*n* = 5)

Sample	Added (μg g^−1^)	Recovery (%)	RSD (%, *n* = 5)
Chicken breast	0.01	87.5	3.8
	0.02	89.1	3.2
	0.03	92.2	4.6
Chicken liver	0.01	93.8	4.1
	0.02	94.4	3.6
	0.03	88.4	3.7

**Fig. 16:**
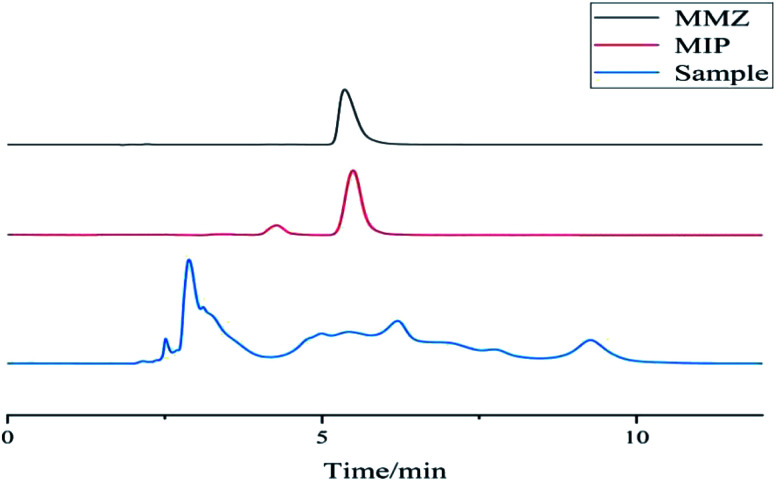
Chromatograms of chicken liver samples before and after solid-phase extraction.

Fig. 16 shows the chromatograms of chicken samples before and after solid-phase extraction. The abovementioned figure shows that the chicken tissue sample has complex components. The sample that was not processed by the MISPE cartridge was directly analyzed by HPLC, and the target component MMZ had interference; the eluate after the MISPE cartridge was subjected to liquid phase analysis. The MISPE cartridge has good enrichment ability. Because the filler in the MISPE cartridge has a binding site with a higher matching degree with the structure of MMZ, it can specifically adsorb MMZ, which has a good enrichment effect and effectively removes interference.

## Conclusions

In this study, the dendritic fiber-type silica material KCC-1 is used as the substrate, and nanosilver is loaded on the surface of the core–shell thiimidazole molecularly imprinted polymer. Characterization and adsorption experiments show that Ag NPs/KCC-1 molecularly imprinted polymer used as the substrate was successfully prepared, and the prepared polymer has a fast adsorption speed, large adsorption capacity, and good specific adsorption performance for methimazole. The use of it as a filler for solid-phase extraction can selectively enrich methimazole in chicken products, which provides a reference method for the separation and enrichment of methimazole and other veterinary drugs.

## Conflicts of interest

There are no conflicts to declare.

## Supplementary Material
